# Is Vitamin D Supplementation an Effective Treatment for Hypertension?

**DOI:** 10.1007/s11906-022-01204-6

**Published:** 2022-06-23

**Authors:** Songcang Chen, Gio Gemelga, Yerem Yeghiazarians

**Affiliations:** Division of Cardiology, Department of Medicine, University of California San Francisco, San Francisco, CA 94143 USA

**Keywords:** Vitamin D, Deficiency, Blood pressure, Hypertension, Randomized clinical trial

## Abstract

**Purpose of the Review:**

Results from epidemiological studies suggest that vitamin D (VD) deficiency (VDD) may be a cause of hypertension (HTN). However, the results of randomized clinical trials (RCTs) designed to address the impact of VD supplementation on reducing blood pressure (BP) remain equivocal. To determine whether VD might serve as a beneficial treatment option for a specific subset of hypertensive patients, we performed a stratified analysis of RCT data and addressed problems associated with some methodological issues.

**Recent Findings:**

HTN is caused by multiple factors. VDD may be one of the factors contributing to the development of this disorder. There are more than 70 RCTs that examined the impact of VD supplementation on BP. These RCTs can be classified into four groups based on their respective study populations, including participants who are (1) VD-sufficient and normotensive, (2) VD-deficient and normotensive, (3) VD-sufficient and hypertensive, and (4) VD-deficient and hypertensive.

**Summary:**

Our evaluation of these studies demonstrates that VD supplementation is ineffective when used to reduce BP in VD-sufficient normotensive subjects. VD supplementation for five years or more may reduce the risk of developing HTN specifically among those with VDD. Interestingly, findings from 12 RCTs indicate that daily or weekly supplementation, as opposed to large bolus dosing, results in the reduction of BP in VD-deficient hypertensive patients. Our ongoing research focused on elucidating the mechanisms of VDD-induced HTN will ultimately provide evidence to support the development of etiology-specific prevention and treatment strategies focused on HTN in the VD-deficient population.

## Introduction

Uncontrolled hypertension (HTN) is a major risk factor for stroke, cardiovascular disease, renal failure, and increased maternal mortality [1]. Although several different types of antihypertensive medications are currently available [2], ~50% of hypertensive patients in the USA have blood pressure (BP) that is poorly controlled. Furthermore, approximately five million patients with HTN are resistant to therapy [3]. A recent publication in the *Journal of the American Medical Association* [4••] reported that in 2017–2018, only 43.7% of adults diagnosed with HTN exhibited adequate BP control (age-adjusted rate); this represented a decline from 53.8% reported for the years 2013–2014. Among the major problems limiting effective treatment of this disorder, more than 95% of these patients have been diagnosed with essential HTN of unknown cause with no etiology-specific therapies. The less effective yet lifelong therapies available for these patients have become a severe burden for our society [5]. Thus, efforts to identify causal factors and therapeutic targets are urgently needed in promoting the development of etiology-specific prevention and treatment of HTN.

Vitamin D deficiency (VDD) is commonly defined as serum 25-hydroxy vitamin D (25[OH]D) levels <20 ng/ml [6, 7]. The well-characterized sequelae of VDD include nutritional rickets in children, osteomalacia in women, and increased severity of fractures secondary to falls in the elderly. Over the past 20 years, large-scale cross-sectional and cohort analyses, as well as Mendelian randomization and prospective studies, have demonstrated that serum 25[OH]D levels are inversely related to elevated BP, HTN, and adverse cardiovascular events [8]. These findings are supported by the results of studies using genetic and nutrient-depleted VD-deficient animal models that display hypertensive phenotypes [9]. Collectively, the results of these studies suggest that VDD may also contribute to human HTN and that VD supplementation may be effective means for its prevention and treatment. Well-designed RCTs are powerful tools that might be used to assess the contributions of VD in HTN. Although RCTs are currently considered the gold standard, studies that are poorly designed, conducted, analyzed, and reported can provide highly misleading results. When designing RCTs to address the impact of VD, which has been identified as a threshold nutrient [10] (described further below), clarity with respect to the participants’ baseline BP and serum 25[OH]D levels, as well as the specific dosing regimens is critical to their ultimate success (see below in detail).

At this time, findings are available from more than 70 RCTs that were designed to evaluate the role of VD in reducing BP. These can be classified into four groups based on baseline levels of serum 25[OH]D and BP in selected study populations; of note, several of the earliest RCTs are without serum 25[OH]D data. As a group, these studies include participants that are (1) VD-sufficient and normotensive, (2) VD-deficient and normotensive, (3) VD-sufficient and hypertensive, and (4) VD- deficient and hypertensive. We have also re-evaluated these findings based on the recent changes in the guidelines used to diagnose HTN. While the new guidelines, which have reduced the threshold for diagnosing HTN [11], may benefit some patients at high risk of developing cardiovascular disease, most individuals are identified as hypertensive at a BP of 140/90 mmHg or greater [12]. Many of the clinical trials discussed in this review were completed before the release of the new guidelines. Thus, for this review, “normotensive” denotes BP < 140/90 mmHg and “hypertensive” refers to BP ≥ 140/90 mmHg.

## VD Supplementation is Ineffective in Reducing BP in VD-Sufficient Normotensive Subjects

In 1998, Krause et al. [13] used ultraviolet B (UVB) irradiation to treat a group of patients with otherwise untreated essential HTN and VDD. The treatment resulted in a 162% increase in serum levels of 25[OH]D and a significant reduction (6 mmHg) in both systolic and diastolic BP (SBP/DBP). While this finding has led some investigators to perform clinical trials using VD to treat this specific cohort of hypertensive patients, other investigators have gone forward to examine the use of VD to reduce BP in the general population. This direction was largely based on the theory that VD could suppress basal expression of the endogenous hormone, renin [14]. Since that time, many lines of evidence have emerged that suggest that a modest increase in renin levels has little to no impact on VDD-associated HTN [8, 9].

In contrast to exogenous BP-reducing drugs (for example, the renin inhibitor, aliskiren), VD is synthesized in skin exposed to sunlight and can also be obtained from dietary sources (e.g., milk). VD is a well-known threshold nutrient, meaning that physiological responses (e.g., promoting calcium absorption) are dose-dependent at low concentrations; once a threshold value is reached, higher levels of the given nutrient promote limited beneficial effects [10, 15••, 16••]. Thus, it will be critical to have a clear understanding of endogenous VD levels in participants selected for this type of study. One is unlikely to see a great benefit from VD supplementation of normotensive study participants if their baseline serum 25[OH]D levels are already above the threshold level. These observations can explain the negative outcomes from many of the RCTs that demonstrated no effect of VD supplementation on BP in the general population, including those who are normotensive and VD- sufficient [8, 17–19].

The ongoing vitamin D and Omega-3 HTN trial (VITAL HTN; NCT01653678) was designed to examine long-term VD supplementation (2000 UI daily for five years) as a means to prevent HTN in participants aged ≥ 50 years who presented with normal BP. VITAL researchers have already published one report [20], stating that VD supplementation did not reduce the incidence of cardiovascular events among the participants who presented with adequate mean levels of VD (only 12.7% of participants were VD-deficient). These preliminary results suggest that VD supplementation is unlikely to have a significant impact on the incidence of HTN in participants who were VD-sufficient at baseline.

## A Five-Year Trial of VD Supplementation May Reduce the Risk of Developing HTN in Vulnerable Normotensive Populations Presenting with VDD

Low serum VD levels have been associated with an increased risk of developing HTN [8, 9]. Thus, VD supplementation should reduce the incidence of HTN in susceptible subjects with VDD and normotension at baseline. Because HTN is an age-dependent, chronic disease with a long induction period, it may be essential to provide long-term VD supplementation to this target treatment group. Recent advances from large RCTs suggest that VD supplementation should be provided over a five-year period in order to determine its effects on the incidence of chronic diseases (e.g., HTN) [15••, 16••]. Thus, the negative results from previous studies in which VD supplementation was time limited (≤18 months) may need to be re-evaluated, as the study period was not long enough to record a sufficient number of events [21–27].

Results from a recent clinical trial revealed that two years of VD supplementation at 800 IU and 2000 IU per day reduced mean SBP by 3.94 and 2.75 mmHg, respectively, in a VD-deficient cohort of patients ≥60 years of age [28•]. Unfortunately, this study did not include a placebo group, and thus, the findings presented do not permit us to conclude that VD supplementation effectively reduces BP. Future RCTs that include placebo groups should determine whether a similar or more reduction in BP can achieved with these doses of VD over five years in vulnerable VD-deficient patients. These findings would be of substantial clinical significance because every 10-mmHg reduction in SBP has been associated with reduction of 20% in major cardiovascular disease events, 17% in coronary heart disease, 27% in stroke, 28% in heart failure, and 13% in all-cause mortality [29]. It is critical to note that there were no statistically significant differences between the responses to 800 IU and 2000 IU [28•]; this observation suggests that the maximum impact of VD on reducing BP in a normotensive population may be achieved via a regimen of 800 IU per day for two years or longer. Subsequently, the same group published the results of the DO-HEALTH RCT which demonstrated that daily supplementation with 2000 IU or control (i.e., a lower dose of VD) over three years reduced SBP by 8.6 and 7.9 mmHg, respectively, in hypertensive elderly cohort with VD insufficiency [30]. Since all participants were allowed to take 800 IU VD daily in the DO-HEALTH RCT [31], one assumes that the “no VD” control group [30] was taking this lower dose. Reductions in BP among those treated with 2000 IU VD per day were no different from the responses of the control group, suggesting that daily 800 IU of VD for three years provided the maximum benefit in reducing BP in elderly patients with both HTN and VD insufficiency.

At baseline, BP is regulated by interactions between genetic, epigenetic, and environmental factors that maintain a stable balance between vasocontraction and vasodilation [8]. While VDD may also result in elevated BP in those <40 years of age, these younger individuals may have the capacity to maintain normal BP and vascular tone due to sufficient compensatory mechanisms (e.g., endothelial nitric oxide synthase [eNOS]-dependent signaling pathways). Thus, younger people with VDD may not develop HTN [32].

## VD Supplementation has a Modest Effect on Some Hypertensive Patients Who are VD-Sufficient

Many different factors including single gene mutation (e.g., Liddle syndrome) and environmental factors (e.g., high-salt diet) can cause HTN. VDD is potentially one of the causal factors for HTN. Unlike nutritional rickets caused by a single factor (VDD) and cured by VD repletion, VD supplementation is unlikely to be an effective treatment for all types of HTN caused by multiple factors. For example, Larsen et al. [33] treated 112 hypertensive patients with 3000 IU VD or placebo daily for 20 weeks. In this study, supplementation with VD resulted in increased serum levels of VD levels but had no significant impact on BP evaluated over a 24-h period. However, a post hoc analysis of 92 study participants who presented with VDD or VD insufficiency at baseline revealed dramatic reductions in SBP/DBP specifically in this subgroup. These results suggest that patients who develop HTN secondary to other causes other than VDD may not benefit substantially from VD supplementation.

As we noted earlier in this review, VD provided at levels above a specific threshold may not lead to additional beneficial effects under normal physiologic conditions. However, during the development of some types of HTN, the threshold level for VD can be increased, a phenomenon in which enhanced VD signaling acts as a negative feedback hormonal regulator to inhibit an excessively high BP level. Animal studies have revealed that administration of VD or its analogs can result in modest, but significant reductions in BP in spontaneously hypertensive rats (SHRs) [34, 35] and in cases of angiotensin II (Ang II)-induced HTN in mice [36], while they have no impact on high-salt induced HTN [37]. These findings suggest that boosting VD signaling may result in reductions in BP in certain types of HTN with normal VD levels at baseline and that VD exerts its antihypertensive role partly via its capacity to reduce Ang II-induced vasocontraction and enhance atrial natriuretic peptide-induced vasodilation (Chen S et al. submitted). While an appropriate dose and dose interval of VD may result in reductions in BP in some, but not all VD-sufficient hypertensive patients, the results from the clinical trials that recruited hypertensive cohorts who are VD sufficient may not be fully consistent.

## VD Supplementation Reduces BP in Hypertensive Patients with VDD

An ideal RCT designed to determine whether VD can be an effective treatment for HTN should target hypertensive patients with VDD. The experimental group should be treated with a constant, non-intermittent physiological dose of VD, while the controls should be treated with either placebo or VD at an ineffective low dose. To date, more than 10 trials developed using this design (including ours in which the key methods and findings were described [8]) have revealed that daily supplementation with VD (800–4000 IU), VD analogs, or UVB radiation restored serum 25[OH]D levels and resulted in a significant reduction in BP in VD-deficient hypertensive subjects [13, 33, 38–44]. These findings are consistent with the results of two earlier RCTs that also found that daily administration of a VD analog could reduce BP in hypertensive patients in which 25[OH]D levels were not known [45, 46]. Another two RCTs demonstrated that weekly doses of VD resulted in effective reductions in BP in hypertensive patients with VDD [47, 48]. Despite the comparatively small sample sizes in these trials, these outcomes consistently demonstrate that daily or weekly administration of appropriate doses of VD or its analogs can significantly reduce BP in VD-deficient hypertensive populations. These data provide the rationale for studies designed to elucidate the cellular and molecular mechanisms underlying VDD-induced HTN. The results of these mechanistic studies will provide a molecular basis and evidence to support large RCTs in which VD supplementation is used for etiology-specific prevention or treatment of HTN. Toward this end, our group has shown that cell-specific deletion of the VD receptor in vascular smooth cells results in HTN which may be due to a concomitant increase in the expression of modulatory calcineurin-interacting protein 1 (Chen S et al., submitted). The results of this study suggest that VD signaling in vascular smooth muscle cells may play a critical role in the pathogenesis of VDD-induced HTN.

Some of the trials in this group reported negative results, many of which might be attributed to problems with the study designs. The use of intermittent large bolus doses [49–53] leads to wide fluctuations in circulating VD levels and does not provide the steady and effective dose required by the vascular system [15••, 54–56]. Large boluses of VD may even have a toxic effect on the vascular walls (discussed below).

## VD Supplementation Via Intermittent Large Bolus Doses has no Impact on BP in Hypertensive Patients with VDD

VD is an essential nutrient that supports bone health [7]. While administration of VD (e.g., 800–2000 IU daily) reduces the risk of fractures secondary to falls in the elderly [57], monthly or yearly administration of large bolus doses (e. g., 60,000–100,000 IU monthly or 300,000–500,000 IU annually) has no effect or may even increase the risk of fracture [58–62]. By contrast, daily doses of more appropriate concentrations of VD (e.g., 800–4000 IU daily) or its active forms are effective antihypertensive treatments for patients with both HTN and VDD [13, 33, 38–43]. Several trials have examined the impact of intermittent large bolus doses (e.g.,100,000 IU VD at 1–3 times per year) and reported that this regimen does not reduce BP in VD-deficient patients with HTN [49–53], most notably in those >70 years of age [49, 51, 52]. Interestingly, two previous small RCTs reported that large bolus doses of VD reduced SBP in patients with type 2 diabetes (T2D) and HTN after 8 weeks [63, 64], but not at 16 weeks of treatment [64]. The transient hypotensive role of VD administered in this fashion may be related to its toxic effect on patients with T2D and HTN who frequently exhibit larger blood volumes due to high serum glucose levels. Consistent with this interpretation, we found administration of high-dose VD to rats led to a significant increase in daily urine volume and a decrease in body weight (Chen S et al., unpublished data).

At this time, we have no clear understanding of the mechanisms underlying the vascular toxicity mediated by large bolus doses of VD or the lack of response among hypertensive patients with VDD. Several lines of evidence may be introduced that might clarify this scenario. Heaney et al. [54] found that a bolus injection of 100,000 IU VD in a group of healthy adults (n = 30) led to the elevation of plasma mean VD levels of 521 pmol/L. This concentration, which was 100-fold higher than the basal level evaluated over the previous 24 h, returned to near baseline in seven days. The injection resulted in a slow rise in serum 25[OH]D; these levels reached a peak at 515 nmol/L at seven days and returned to baseline (~68 nmol/L) after about four months. The active form of VD, 1,25-dihydroxyvitamin D (calcitriol) is synthesized by 25-hydroxylation of VD in the liver and subsequent 1-hydroxylation in the kidney and can also be generated locally in specific tissues [8]. VD-25-hydroxylase (also known as sterol 27-hydroxylase) is expressed in vascular endothelial cells [65, 66]; 25[OH]D 1-alpha-hydroxylase is expressed in both vascular endothelial and smooth muscle cells [67, 68]. The high concentration of VD introduced by bolus injection may result in the overproduction of calcitriol specifically in vascular endothelial and smooth muscle cells. In several animal models (e. g., pigs, rats, goats, and mice), large doses of VD can induce an osteoblastic phenotype in vascular smooth muscle cells that ultimately results in vascular calcification [69]. Elderly individuals (≥70 years old) frequently suffer from vascular stiffness; isolated systolic HTN is believed to be the direct result of vascular stiffness and calcification. Thus, the high doses of VD (e. g., 100,000 IU) administered in several of the aforementioned trials [49, 51–53] may actually promote vascular calcification, thereby counteracting any of its beneficial antihypertensive effects. Furthermore, while two studies demonstrated that large bolus doses of VD resulted in serum 25[OH]D levels that were similar to those reported in response to daily doses of VD, the levels obtained in response to the large bolus doses dropped significantly at the completion of the trials [70, 71]. Given that (1) vascular tissue cells can synthesize calcitriol and (2) circulating 25[OH]D levels do not reflect the full extent of VD activity in target tissues [72, 73], serum VD levels have been considered more important than serum 25[OH]D for the evaluation of VD activity in target tissues [55, 56]. Intermittent large bolus doses of VD that result in a rapid rise in serum VD levels followed by a similarly rapid return to basal levels may also contribute to the observed lack of effect of VD supplementation in several trials that included VD-deficient hypertensive populations. Finally, as described above [54], intermittent large bolus doses of VD produce wide fluctuations in circulating VD and 25[OH]D levels. These levels may change the physiological functioning of VD so that it will have no effect on the prevention of overall mortality due to fractures and falls among the elderly [74]. This treatment regimen may not be beneficial in patients with VDD-induced HTN because its impact is dramatically different from that of the daily dose schedule (e.g., 800–4000 IU per day) that generates a steady, more physiologic increase in serum VD and 25[OH]D levels over a longer period [75].

Further research will be required to elucidate the underlying mechanisms via which large bolus doses of VD promote vascular calcification. Additional studies will also be needed to determine why VD administered in this fashion either has no therapeutic efficacy or a detrimental impact on VDD-induced HTN. Nevertheless, a consensus of recent reviews has concluded that a constant physiological dose of VD results in steady levels of serum VD and 25[OH]D with optimal benefits. By contrast, intermittent large bolus doses of VD do not achieve steady or effective levels of VD and 25[OH]D and should not be used in these treatment regimens [15••].

## Re-Evaluation of Meta-Analyses of Several RCTs

Meta-analyses aim to provide a more precise estimate of the effects of a specific situation or intervention as they can increase sample size and power by a combined review of primary studies with similar populations, controls, interventions, and outcomes. However, it has become clear that administration of VD to subjects in each of the four different aforementioned groups (i.e., VD-sufficient and normotensive, VD-deficient and normotensive, VD-sufficient and hypertensive, and VD-deficient and hypertensive) results in dramatically different outcomes. Furthermore, large intermittent bolus doses of VD are ineffective in reducing BP, even in hypertensive VD-deficient patient cohorts. Thus, pooling data from the four different groups will introduce a considerable amount of heterogeneity. Likewise, meta-analyses that do not consider the specific dosing regimens will also provide mistaken support for the null hypothesis. While none of the published meta-analyses designed to evaluate the role of VD supplementation in reducing BP have provided uniformly positive results, several recent reviews do conclude that VD supplementation can reduce BP in VD-deficient hypertensive patients [76, 77]. The conclusions from these two meta-analyses [76, 77] are supported by evidence from seven RCTs [33, 40, 42, 63, 64, 78, 79] that included 242 participants with an average age of 58.4 years who were recruited to the VD supplementation arm with mean baseline SBP/DBP ± SD of 134.7 ± 6.6/79 ± 4.9 mmHg. The administration of VD for 8–24 weeks reduced mean SBP/DBP by 5.5/2.6 mmHg (95% CIs, 5.2–5.8/2.4–2.8). All seven trials included patients undergoing treatment with HTN medication; however, the percentage of patients undergoing treatment and the types of HTN medication used varied widely. Co-administration of a calcium channel blocking agent had relatively little impact on the antihypertensive effect of VD [40]. However, VD supplementation alone resulted in minor and insignificant reductions in BP compared with the administration of placebo in one trial in which 85–90% and 70–85% of the participants were treated with angiotensin-converting enzyme/Ang II receptor inhibitors or diuretics, respectively [78]. As we noted above, VD exerts its antihypertensive effects partly via reductions in enhanced Ang II-induced vasoconstriction and recovery of impaired atrial natriuretic peptide-induced vasodilation. Administration of agents that block increases in Ang II and its downstream signaling molecules and/or the use of diuretics will mask the antihypertensive effects of VD in VDD hypertensive patients. Although all of these agents can reduce BP in these patients, VD may be an effective treatment that is also etiology-specific. By contrast, Ang II signaling blockers and diuretics are non-specific treatments that require lifelong administration. Patient age and ethnicity do not seem to have an impact on the results reported in these trials.

Other meta-analyses [80, 81] have not focused on problems with study methodology and have thus generated data that are not consistent with those that document the benefits of VD administration. One such study was performed by Beveridge et al. [80]. This meta-analysis included 46 clinical trials that were selected based on their use of VD supplementation for a minimum of 4 weeks and reported BP measurements. In 17 of these trials, participants had a mean baseline SBP ≥140 mmHg (based on findings shown in Fig. 2 and Table 1 of this publication); in seven of these trials, administration of VD or VD analogs results in a significant reduction in BP [41, 43, 45, 46, 63, 64, 82]. Of note, one trial reported that VD had no effect overall, but significantly reduced BP in the hypertensive subgroup with baseline 25[OH]D levels less than 32 ng/ml [33]. Another trial included in the meta-analysis reported that, when combined with several antihypertensive medications, administration of both VD and placebo resulted in significant decreases in BP [83]. Among the eight trials that reported no response to VD, one used a lower daily dose (400–600 IU) [84] and five administered large bolus doses [49, 51–53, 50]. Four studies in the latter group administered 100,000 IU VD to the elderly or patients with resistant HTN at intervals of 1–3 times in one year [49, 51–53] which is a treatment regimen that does not achieve steady or effective levels of VD and 25[OH]D [15••]. The large bolus dosing strategy used in each of these five trials may have led to the overall negative results. However, the authors of the meta-analysis did not specifically analyze treatment regimens and baseline VD levels in the relevant subgroup. Instead, they combined the subgroup data with those from the other 29 trials that recruited normotensive participants, from which they had concluded previously that VD supplementation did not affect BP in normotensive cohorts [85]. Thus, it is no surprise that pooling the data from all 46 trials, regardless of VD and hypertensive status revealed no significant reduction in BP from VD or its analogs.

Of note, at the end of the Results section of this meta-analysis and review, Beveridge et al. [80] reported that they analyzed data from a small group of patients (n = 60) with HTN and severe baseline VDD (25[OH]D at <10 ng/mL and parathyroid hormone >217 pg/ml). They stated that administration of VD was without benefit in these patients, but no references to primary studies were provided. Thus, we have no way of knowing whether these 60 patients received daily or intermittent large bolus doses of VD. Although we understand that this meta-analysis and review was published seven years ago before the data from the most recent RCTs were available, it is helpful to highlight the potential for misinterpretation that can be introduced when one pools data from heterogeneous trials without a clear assessment of potential errors in methodology. In this case, the meta-analysis led to a false conclusion regarding VD and its effectiveness in reducing BP. It is important to correct these conclusions as the field progresses.

## Summary

Unlike pharmaceutical agents (e. g., prazosin or captopril), VD is an endogenous nutrient with a specific threshold that limits its therapeutic efficacy in lowering BP. It is critical to recognize that VD is not a panacea as it is not effective in reducing BP in the VD-sufficient normotensive population. However, an appropriate VD regimen (e. g., 800–4000 IU per day) can reduce BP in patients diagnosed with some types of HTN, most notably HTN secondary to VDD. Long-term VD supplementation (five years) may also reduce the risk of developing HTN in VD-deficient susceptible populations. Our ongoing studies focused on the molecular and cellular links between VDD and HTN will support the development of etiology-specific therapeutics for and prevention of HTN in this critical patient subgroup.
